# Coprological detection of equine nematodes among slaughtered donkeys (*Equus asinus*) in Kaltungo, Nigeria

**DOI:** 10.14202/vetworld.2019.1911-1915

**Published:** 2019-12-07

**Authors:** Tobias Nnia Egbe-Nwiyi, Bura Thlama Paul, Ajuji Chungsyn Cornelius

**Affiliations:** 1 Department of Veterinary Pathology, Faculty of Veterinary Medicine, University of Maiduguri, Bama Road, Maiduguri 600230, Nigeria; 2 Veterinary Teaching Hospital, Faculty of Veterinary Medicine, University of Maiduguri, Bama Road, Maiduguri 600230, Nigeria

**Keywords:** detection, donkey, egg per gram, Gombe, nematodes, Nigeria

## Abstract

**Aim:**

This study aimed to investigate the prevalence and intensity of nematode infection among slaughtered donkeys in Kaltungo, Nigeria.

**Materials and Methods:**

A total of 72 fecal samples were examined by salt flotation and the modified McMaster fecal egg count technique to morphologically identify nematodes eggs and determine their egg per gram (EPG) outputs.

**Results:**

Out of a total of 72 (100%) donkeys sampled, 36 (50%) tested positive, but the prevalence of nematodes was independent of the age, sex, and breed of donkeys (p>0.05). Among the four species of nematodes identified in single and mixed infections, *Strongylus* spp. (27.8%) and *Dictyocaulus arnfieldi* (13.9%) were the most prevalent followed by *Strongyloides westeri* (5.6%) and *Trichonema* spp. (5.6%). Infected donkeys had moderate overall mean EPG (801.39±611.3) with no statistical differences between age groups and sexes (p>0.05), but means of EPG were significantly higher (p<0.05) in Duni (1026.92±719.55) than Idabari (673.91±514.75). Light EPG count was recorded among 63.9% of infected donkeys, while 16.7% and 19.4% had moderate and severe infections, respectively.

**Conclusion:**

The prevalence and importance of equine nematodes were discussed in connection to their epidemiology and control. Furthermore, the preponderance of light infection may suggest that donkeys in this environment developed resistance to nematode infection and are potential reservoirs for other equines.

## Introduction

The domestic donkey (*Equus asinus*), a member of the family *Equidae*, was first domesticated in Africa about 5000 years ago but now has a global distribution [1]. The global population of donkeys is 44 million [2] and 98% occur in semi-arid zones of Africa [1,3], where they are used for working, breeding, milking, and meat purposes [4]. In Nigeria, the estimated 1.4 million donkeys serve as essential sources of farm power, transportation, and protein in some rural parts of Nigeria [5].

A diverse group of helminth species including the small Strongyles (*Cylicocyclus elongatus* and *Cyathostomum pathratum*), large Strongyles (*Strongylus vulgaris*, *Strongylus equinus*, and *Strongylus edentatus*), *Triodontophorus* species, anoplocephalid tapeworms, ascarid (*Parascaris equorum*), *Oxyuris equi*, *Trichonema* species, and *Dictyocaulus arnfieldi* has been identified in donkeys [6]. Nematode infection of donkeys affects their health, productivity, and working capacity, in addition to serving as sources of pasture contamination for domestic horses. Nematode parasites are responsible for the poor condition, reduced efficiency, poor reproductive performance, retarded growth, and mortality of horses, donkeys, and mules [3]. Among these, Strongyles are the most common pathogenic species found globally [3,7,8]. Equine strongylosis may be due to the migratory larvae or adult parasites in the intestine. The presence of large Strongyles in the gut of donkeys leads to poor health and dysfunctions such as colic while the larvae of small Strongyles penetrate the large intestine and cause acute diarrhea, colic, and occasionally death due to toxic shock [6,9]. Equine lungworm, *D. arnfieldi* is also found worldwide but rarely causes clinical disease in donkeys even though it is frequently associated with respiratory signs in horses and mules [10]. The prevalence of nematodes in donkeys ranges from 50% to 100%, depending on ecological factors and standard practices [11]. The previous reports on nematodes of the donkey in Nigeria indicated a prevalence of 98% in the northeast [8] and 78% in the northwest [12].

Parasitic diseases threaten the population of donkeys in Nigeria due to the traditional husbandry system. Under the current pastoral nomadism and smallholder practice in Northern Nigeria, donkeys are grazed beside cattle and sheep, exposing them to infection with helminth parasites. Moreover, there are no recognized herd health program targeting donkeys in Nigeria, and their infection with nematodes is poorly understood.

This study aimed to describe the frequency and intensity of nematode ova among slaughtered donkeys in Kaltungo Local Government Area to elucidate the extent of pasture contamination and risk of infection for domestic horses.

## Materials and Methods

### Ethical approval

No ethical approval required for this study because samples were collected from slaughtered animals.

### Study area

Kaltungo is a Local Government Area in the southern part of Gombe State situated within latitudes 9.8423 N and 11.3885 E. The climatology of Kaltungo is characterized by a rainy season from April to October (mean rainfall of 900 mm) and a period of the dry season from November to March, marked by harsh hot winds blowing from the Sahara. The main occupation of inhabitants is crop and livestock production, and donkeys are a part of household livestock for meat and fieldwork.

### Study population and sampling

Donkeys examined in this study were local breeds slaughtered for human consumption in communities of Kaltungo. The age of selected donkeys was determined by dentition [13] and grouped as young (<3 years) or adult (above 3 years). The coat color and morphometric features of donkeys determined various breeds [5,14]. A total of 72 fecal samples collected per rectum from slaughtered donkeys between January and June 2018. Relevant data, including sample number, age, sex, breed, and date of sample collection, were recorded in a casebook.

### Laboratory analysis

Fecal samples preserved in 5% formalin were stored and examined to detect nematode eggs at the Diagnostic Laboratory, University of Maiduguri Veterinary Teaching Hospital. The saturated sodium chloride (400 g of table salt/L of distilled water) floatation technique was used to identify nematode eggs based on their size, shape, color, content (embryo/larvae), and absence of operculum [15-17]. The status of nematode infections was also initially considered semi-quantitatively on a 5-point scoring system; absence of eggs (−), few eggs (+), several (++), many eggs (+++), and very many eggs (++++) in the whole slide [18,19]. The fecal egg counts (FECs) were determined using a previously described modified McMaster FEC technique to estimate the mean and total nematode egg per gram (EPG) outputs among the infected donkeys [16]. The EPG of infected donkeys was classified as light (50-799 eggs), moderate (800-1200 eggs), and severe (over 1200 eggs) [15,16].

### Statistical analysis

The summary of laboratory and field data was presented in Microsoft Excel (2016). Descriptive statistics and Chi-square were computed to determine the prevalence of nematode infection among donkeys while the independent t-test statistic was calculated to compare the mean of EPG between age groups, sexes, and breeds of donkeys using the Statistical Package for the Social Sciences (SPSS) software version 22.0 for Windows (SPSS, Chicago, IL, USA). Statistical significance was set at p≤0.05, and the results were presented in tables and graph.

## Results

**Figure-1:**
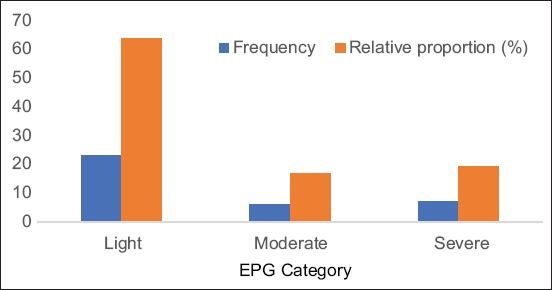
Frequency and relative proportion of egg per gram categories in infected donkeys (n=36).

The results of the present study revealed an overall prevalence of 50% for eggs of equine nematodes. Age-specific incidence was 36.1% and 13.9% in adult and young donkeys, respectively. Breed-specific prevalence was 31.9% in Idabari and 18.1% in Duni, respectively (Table-1). Age, sex, and breed of the donkey did not significantly affect the prevalence of nematodes in this study (p>0.05). FEC revealed that males (747.06±588.6) and adults (701.92±119.116) had a light infection while females (850.00±642.9) and young (1060.0±180.40) had moderate disease, but no statistical difference was observed (p>0.05). However, Duni breed had an average (1026.92±719.55) EPG while the Idabari had light EPG (673.91±514.75) with a statistical difference (p<0.05) (Table-2). This study identified four nematode species from a total of 36 single and mixed infections. Among individual species, *Strongylus* (27.8%) was the most prevalent, followed by *D. arnfieldi* (13.9%), *Strongyloides westeri* (5.6%), and *Trichonema* (5.6%). Out of a total of 36 (100%) infected donkeys, 23 (63.9%), 6 (16.7%), and 7 (19.4%) had light, moderate, and severe nematodes egg counts, respectively (Figure-1). Among mixed infections, the combination of *Strongylus* and *D. arnfieldi* were the most prevalent. Our results further show that 19 (52.8%), 11 (30.6%), and 6 (16.7%) donkeys had a single infection and mixed infection with two and three different species, respectively (Table-3).

**Table-1:** Prevalence of nematodes infection in slaughtered donkeys.

Variables	Number of examined (%)	Number of infected (%)	95% CI	

			L	U
Sex				
Male	37 (51.4)	17 (23.6)	0.15	0.35
Female	35 (48.6)	19 (26.4)	0.18	0.38
Age				
Young	25 (34.7)	10 (13.9)	0.08	0.24
Adults	47 (65.3)	26 (36.1)	0.26	0.48
Breed				
Idabari	41 (56.9)	23 (31.9)	0.22	0.43
Duni	31 (43.1)	13 (18.1)	0.11	0.28
Total	72 (100)	36 (50.0)	0.39	0.61

**Table-2:** Nematode EPG of feces of infected slaughtered donkeys.

Variables	Number of infected (%)	EPG (Mean±SD)	Classification
Sex			
Male	17 (47.2)	747.06±588.6	1
Female	19 (52.8)	850.00±642.9	2
Age			
Young	10 (27.8)	1060.00±570.5	2
Adults	26 (72.2)	701.92±607.4	1
Breed			
Idabari	22 (61.1)	673.91±514.75^a^	1
Duni	12 (33.3)	1026.92±719.55^b^	2
Total	36 (100)	801.39±611.3	2

1,2 represent light and moderate infections, respectively. Means with different superscripts (^a,b^) are significantly different (p<0.05). EPG=Egg per gram

**Table-3:** Relative proportions of nematode species in infected slaughtered donkeys.

Nematode parasites	Frequency	Percentage
*Dictyocaulus arnfieldi*	5	13.9
*Strongyloides westeri*	2	5.6
*Strongylus* spp.	10	27.8
*Trichonema* spp.	2	5.6
*Strongylus* spp.+*Dictyocaulus arnfieldi*	5	13.9
*Strongylus* spp.+*Dictyocaulus arnfieldi*+*Strongyloides westeri*	5	13.9
*Strongylus* spp.+*Strongyloides westeri*	3	8.3
*Strongylus* spp.+*Strongyloides westeri*+*Trichonema* spp.	1	2.8
*Dictyocaulus arnfieldi*+*Strongyloides westeri*	2	5.6
*Dictyocaulus arnfieldi*+*Trichonema* spp.	1	2.8
Total	36	100

## Discussion

This study revealed that out of the total of 72 donkeys examined, 36 (50%) tested positive for one or more species of pathogenic nematode parasites. Furthermore, age, sex, and breed of donkeys investigated in this study had no significant effects on the prevalence of nematode infection (p>0.05). Nematode infection is significant in donkeys due to their impact on health, productivity, and working capacity, in addition to contamination of pasture for domestic horses [20]. The prevalence of nematode infection in donkeys ranges between 50% and 100% depending on ecological factors and management practices [11]. The maintenance of rural donkeys on strict grazing, especially on contaminated pastures, may increase their exposure to nematode infection [2]. Moreover, anthelmintic treatment of donkeys is not practiced by nomadic and local breeders in Nigeria [8].

The spectrum of nematode fauna identified in this study differs from the previous reports on nematode of donkeys in Nigeria [8,12]. Among the four species of nematodes identified in this study, single and mixed infections of *Strongylus* species (27.8%) and *D*. *arnfieldi* 5 (13.9%) were the most prevalent. Strongyles and *Strongyloides* species were previously reported as the most abundant nematodes of donkey within Northern Nigeria [8,12], but, to the best of our knowledge, this is the first report on *D. arnfieldi* from donkey in this region. This finding may be supported by the fact that most infections of donkeys and horses may not always lead to patency due to larval hypobiosis, which interferes with the lifecycle and detection of eggs in the feces of in immunocompetent individuals [15]. It is, therefore, likely that infection may remain undetected until the host immunity is compromised by environment and nutritional stress or concurrent diseases, leading to the reactivation of arrested larvae and patent infections [16].

Moreover, the period of this study corresponded with the peak of dry season when grazing livestock is challenged by feed shortage and harsh weather condition in Kaltungo. Even though *D. arnfieldi* is not very pathogenic in equids, the mature worms may cause respiratory distress due to obstructive bronchitis, edema, and atelectasis, which represent a serious problem [21]. The prevalence of these parasites is epidemiologically crucial in Northern Nigeria due to their capacity to infect mules and horses [10]. The previous studies have reported the current spectrum of nematodes in domestic and working horses within the region [22,23]. All equine nematodes identified in this study have a direct lifecycle, in which adult worms pass eggs in feces to the environment where larvae (L3) emerge to infect the same (autoinfection) or other susceptible hosts and perpetuate infection [23]. Donkeys may be resistant to these nematodes [1] and therefore serve as potential reservoirs of infection and source of pasture contamination for horses [24]. However, the interactions of coinfecting parasites are known to alter host immunity and pathogenic dynamics as well as interhost and interspecies transmission dynamics [25]. As a result of which, malnutrition, concurrent infectious diseases, overwork and neglect may increase the risk of strongylosis in donkeys [11].

FEC is a vital index in the epizootiology of nematodes; it indicates the extent and intensity of parasitism and the importance of pasture contamination in the transmission of parasites [16]. The pathogenicity of Strongyles and other nematodes is related to their fecundity and host resistance [26]. Therefore, the presence of moderate overall mean EPG in this study indicates moderate grade susceptibility of donkeys to single and mixed nematode infections. Many donkeys have been found to cope with high FEC (>3000 EPG) without a significant impact on health or productivity [24]. This study shows that the mean EPG of Duni was significantly higher (p<0.05) than Idabari breed and was also numerically higher in young donkeys compared with their adult counterparts. Age, sex, and breed differences in prevalence and intensity of gastrointestinal parasites were previously reported in Nigeria [27]. In general, younger animals show clinical symptoms with high egg counts during their first challenge but subsequently develop immunity when they reach adulthood under favorable conditions [15]. The high proportion of light infection observed among the infected donkeys indicates low worm burdens or the presence of active protection and suggests that donkeys may serve as reservoir hosts for other susceptible equids in the study area.

## Conclusion

This study is the first report of *D. arnfieldi* in donkeys in Northeastern Nigeria. The high prevalence of nematode ova among donkeys is important in the epidemiology of equine nematodosis due to their reservoir of infection for horses and mules sharing the same pasture. Further studies are required to investigate the overall prevalence, diversity, and clinicopathological features of nematode infections among equines in Northern Nigeria. The establishment of equine health program targeting donkeys and horses to reduce the prevalence and geographical spread of equine nematodes in Nigeria is recommended.

## Authors’ Contributions

TNE conceived the idea of this study, supervised the project, and revised all drafts of the manuscript; BTP conducted laboratory examinations and prepared all drafts of the manuscript; and ACC participated in sample collection and the laboratory analysis of samples. All authors read and approved the final manuscript.
